# Supplementary material for: Identification of photoperiod-induced specific miRNAs in the adrenal glands of Sunite sheep (Ovis aries)
Source: Front Vet Sci. 2022 Jul 22;9:888207. doi: 10.3389/fvets.2022.888207 (PMC9354845; doi:10.3389/fvets.2022.888207)
Supplement: Supplementary file 1 [file Data_Sheet_1.ZIP › Supplementary file/miRDeep2 file.zip!/miRDeep2 file/Novel_Structure/SPLP42A1/Novel_467.pdf]

Provisional ID	:	NC_019459.2_4818
Score total	:	1.4
Score for star read(s)	:	3.9
Score for read counts	:	-3.9
Score for mfe	:	-0.1
Score for randfold	:	1.6
Score for cons. seed	:	
Total read count	:	4
Mature read count	:	3
Loop read count	:	0
Star read count	:	1

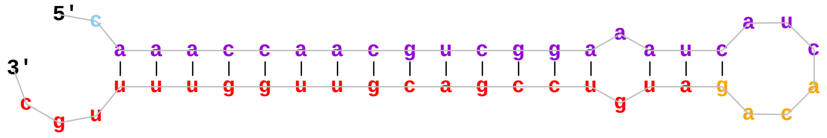

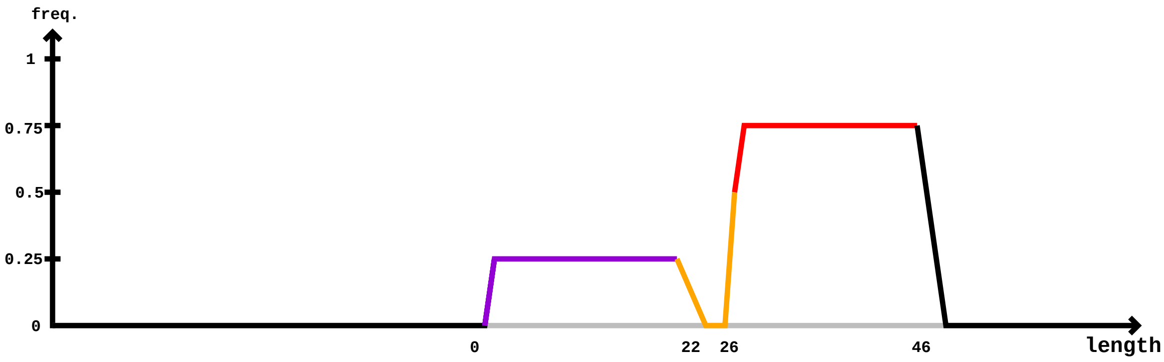

Star **Mature**

```
5'- ugaaaacaaaucauuaucugacguuuuaaacaguauuuagaaacaaaccaacgucggaaaucaucacagauguccgacguugguuuugccuugagaauccauacuaaauuc   -3'    obs
    ugaaaacaaaucauuaucugacguuuuaaacaguauuuagaaacaaaccaacgucggaaaucaucacagauguccgacguugguuuugccuugagaauccauacuaaauuc          exp
    ..(((((...(((.....))).)))))....((((((((....((((((((((((((.(((......))).))))))))))))))....))))......)))))......   reads   mm   sample
    ............................................aaaccaacgucggaaaucauc..............................................   1       0    seq
    .....................................................................auguccgacguugguuuugc......................   1       0    seq
    .....................................................................auguccgacguugguuuugcc.....................   1       0    seq
    ......................................................................uguccgacguugguuuugccu....................   1       0    seq
```
